# Supplementary material for: New Mutants of Epsilon Toxin from Clostridium perfringens with an Altered Receptor-Binding Site and Cell-Type Specificity
Source: Toxins (Basel). 2022 Apr 16;14(4):288. doi: 10.3390/toxins14040288 (PMC9031233; doi:10.3390/toxins14040288)
Supplement: Supplementary file 1 [file toxins-14-00288-s001.zip › toxins-1660190-supplementary.pdf]

Supplementary Materials: New Mutants of Epsilon Toxin from *Clostridium perfringens* with an Altered Receptor-Binding Site and Cell-Type Specificity

Jonatan Dorca-Arévalo, Inmaculada Gómez de Aranda and Juan Blasi

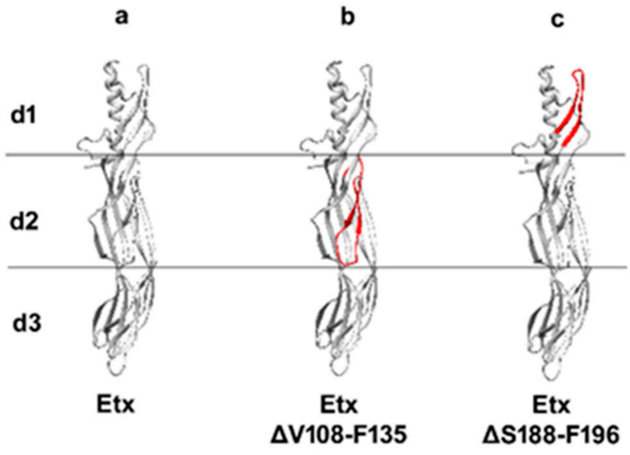

Figure S1. 3D structure of Etx and the location of the deleted portions in each mutant. 3D structure of Etx (a), the mutant Etx-ΔV108-F135 (b), and the mutant Etx-ΔS188-F196 (c). Domain 1, domain 2, and domain 3 correspond to d1, d2, and d3, respectively. See in red the portion deleted in each mutant. In Etx-ΔV108-F135, the deleted portion corresponds to the loop located in domain 2 with the aa sequence VGTSIQATAKFTVPFNETGVSLTTSYSF. In Etx-ΔS188-F196, the deleted portion corresponds to the loop located in domain 1 with the aa sequence SEWGEIPSYLAFPRDGYKF.

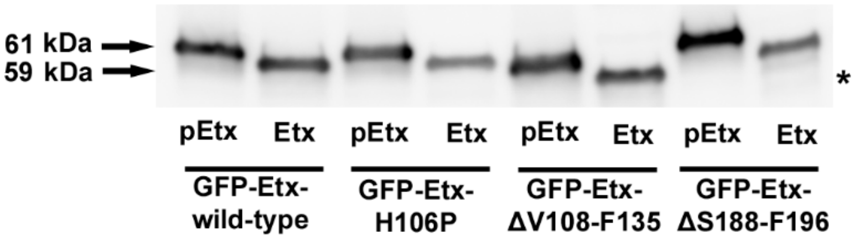

Figure S2. Western blot analysis of purified GFP-Etx and the GFP-Etx mutants. Wild-type GFP-Etx, the mutant GFP-Etx-H106P, the mutant GFP-Etx-ΔV108-F135, and the mutant GFP-Etx-ΔS188-F196 were produced in the prototoxin form in each case (pEtx) or the active form (Etx), with the molecular weight of GFP-Etx-ΔV108-F135 being around 57 kDa (asterisk) compared to the molecular weight of 59 kDa for wild-type GFP-Etx.

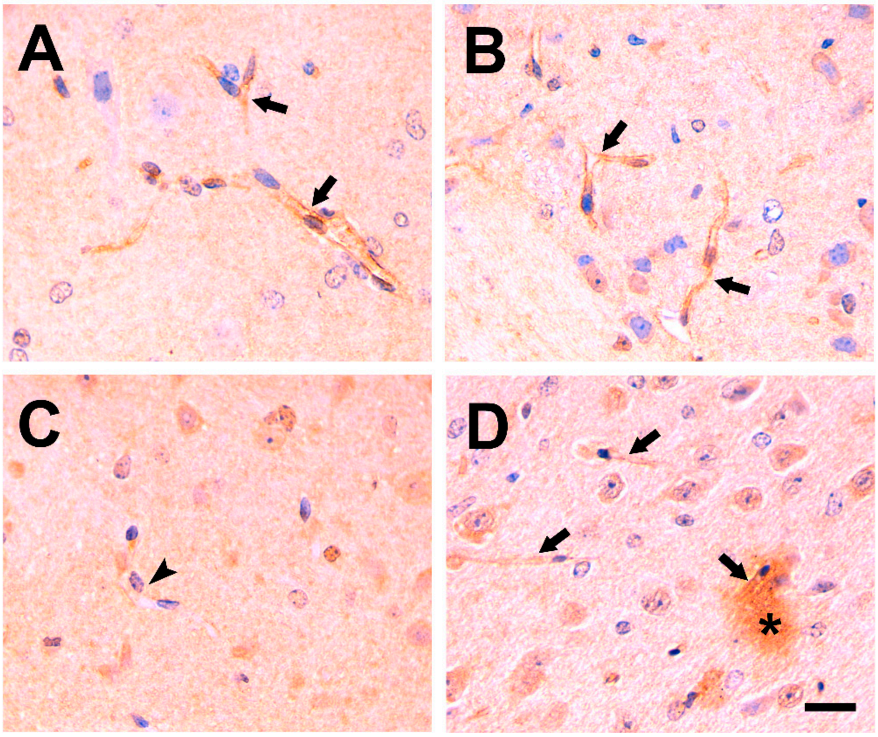

Figure S3. The mutants GFP-Etx-ΔV108-F135 and GFP-Etx-ΔS188-F196 do not cross the blood-brain barrier. Brain sections from mice i.v. injected with GFP-Etx-H106P (**A**), GFP-Etx-ΔV108-F135 (**B**), GFP-Etx-ΔS188-F196 (**C**), or GFP-Etx (**D**) were stained for Etx (brown). GFP-Etx-H106P, GFP-Etx-ΔV108-F135 and GFP-Etx bound to the endothelium (arrows) remaining inside the blood vessel, but GFP-Etx is not only bound to endothelium (arrows in **D**) but also had access to the brain parenchyma attributable to the hability to cross the BBB being distributed by the neuropil (asterisk in **D**). GFP-Etx-ΔS188-F196 did not bind to the endothelium and did not cross the BBB (arrowhead in **C**). Nuclei were stained with hematoxylin. Scale bar, 20 μm.

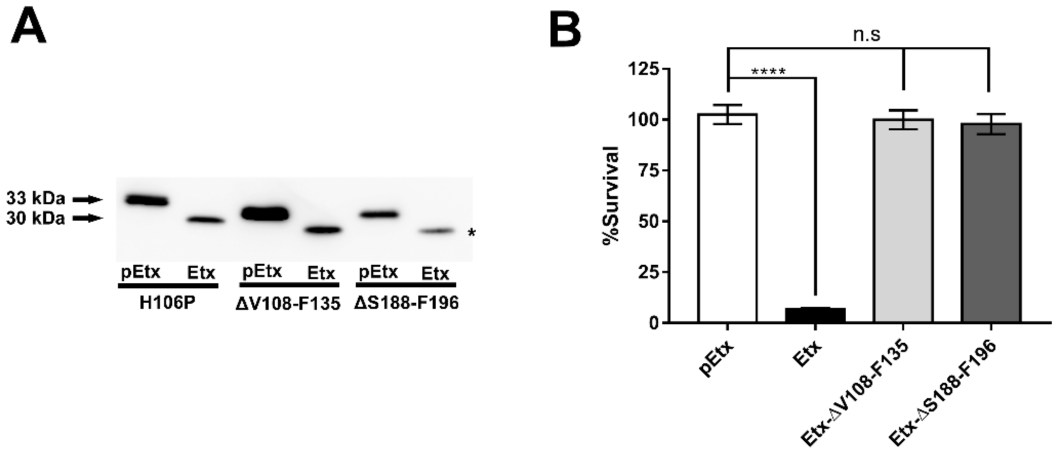

Figure S4. The mutants Etx-ΔV108-F135 and Etx-ΔS188-F196 are non-toxic in MDCK cells. (**A**) Western blot analysis of the inactive (pEtx) or active (Etx) forms of the Etx mutants H106, ΔV108-F135 and ΔS188-F196. Notice the shift in the molecular weight of pEtx-ΔV108-F135 and pEtx-ΔS188-F196 at around 31 kDa and their respective active forms (Etx) at around 28 kDa (asterisk), compared to the 30 kDa for the active form of Etx-H106P, which has a weight similar to that of wild-type Etx [12,31]. (**B**) MTS assays revealed that Etx-ΔV108-F135 (light gray column) and Etx-ΔS188-F196 (dark gray column) were non-toxic in MDCK cells. pEtx (white column) was used as a negative control and Etx (black column) was used as a positive control. In all cases, the cells were incubated with 50 nM of the different forms of Etx at 37ºC for 1 h. (****P < 0.0001), (ns = non-significant).

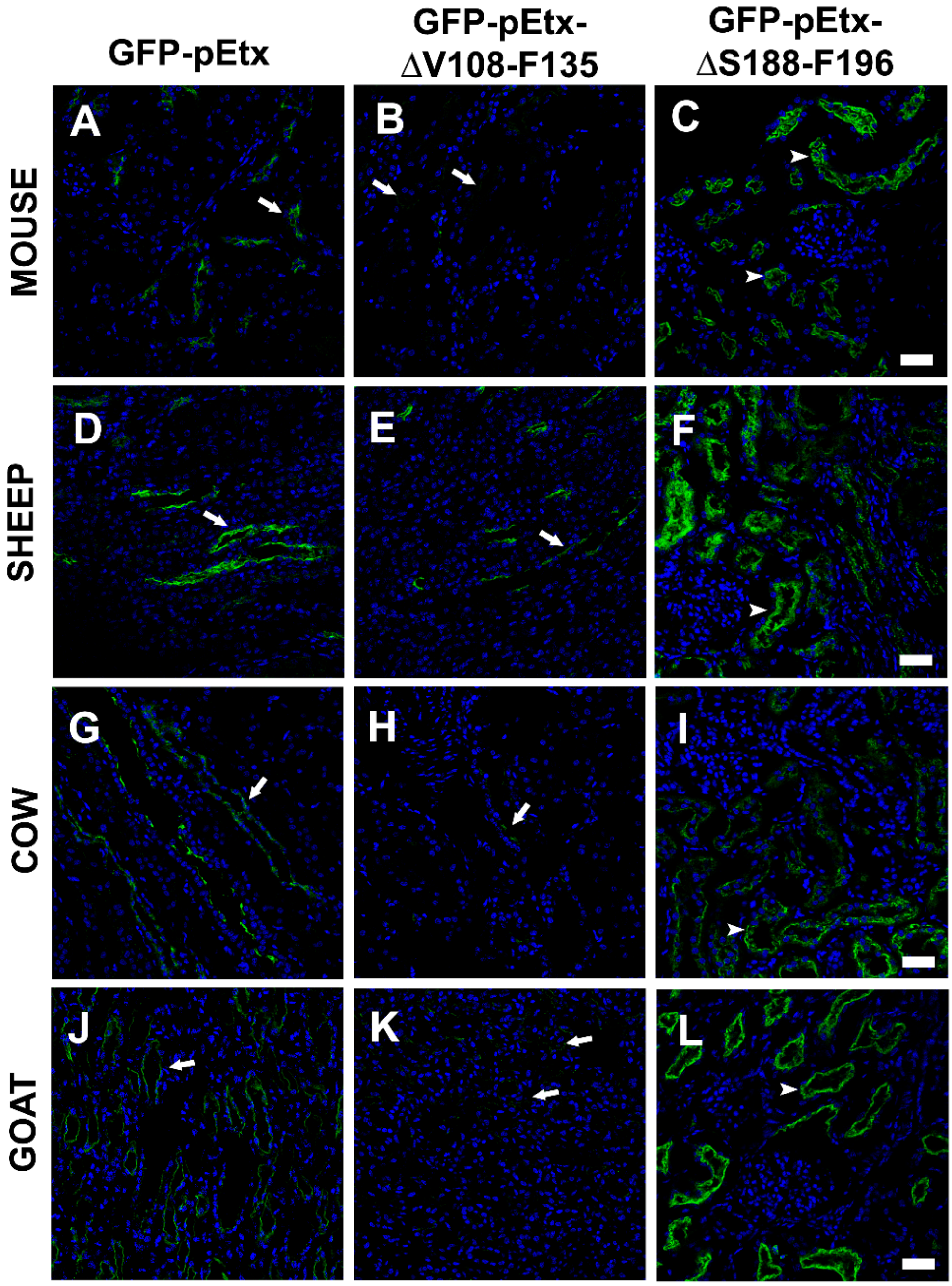

Figure S5. Binding of the GFP-pEtx-ΔV108-F135 and GFP-pEtx-ΔS188-F196 mutants to the kidneys. Confocal images of kidney sections from mouse (**A**-**C**), sheep (**D**-**F**), cow (**G**-**I**) and goat (**J**-**L**) incubated with GFP-pEtx (**A**, **D**, **G** and **J**), GFP-pEtx-ΔV108-F135 (**B**, **E**, **H** and **K**) or GFP-pEtx-ΔS188-F196 (**C**, **F**, **I** and **L**). Note the binding of GFP-pEtx to the distal tubules in all the species tested (in green, arrows in **A**, **D**, **G** and **J**) and a strong decrease in the binding of GFP-pEtx-ΔV108-F135 to the distal tubules (in green, arrows in **B**, **E**, **H** and **K**). GFP-pEtx-ΔS188-F196 does not bind to the distal tubules in any of the species, but recognizes the proximal tubules in all the species tested (in green, arrowheads in **C**, **F**, **I** and **L**). Nuclei were stained with TO-PRO-3 (blue). Scale bar, 40 μm.

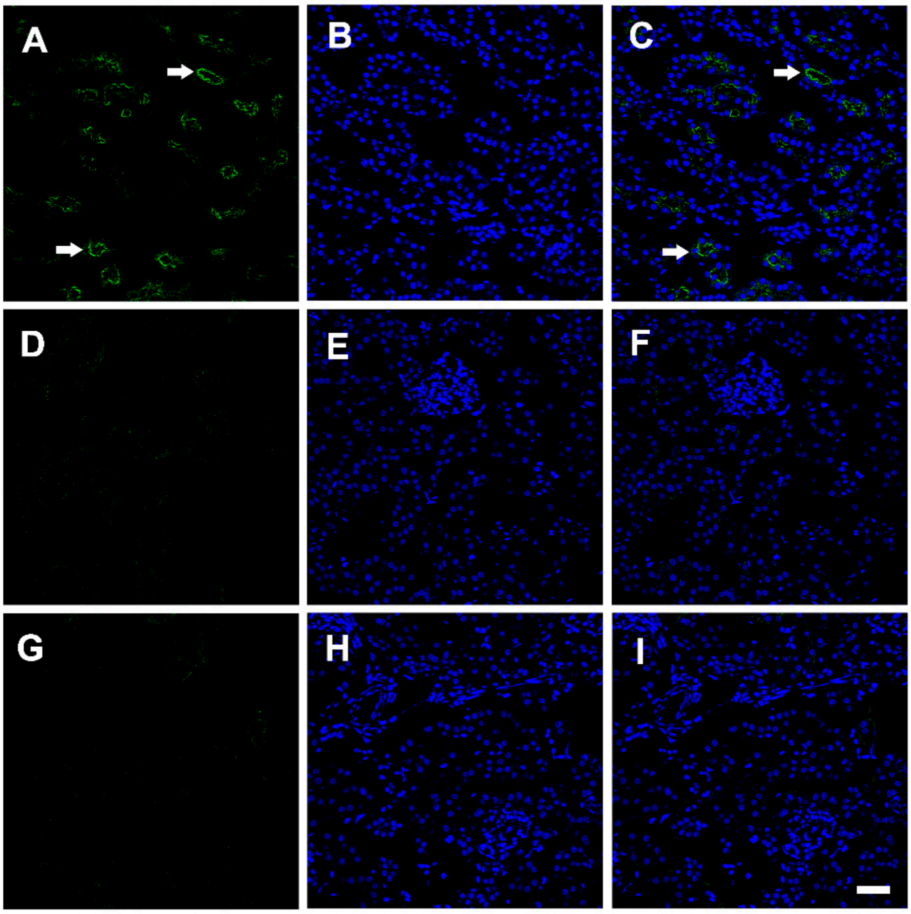

Figure S6. Specific binding of the mutant GFP-pEtx-ΔS188-F196 to the proximal tubules of the kidneys. Confocal images of mouse kidney incubated with the mutant GFP-pEtx-ΔS188-F196 (**A**-**C**), co-incubated with the unlabeled pEtx-ΔS188-F196 in excess and GFP-pEtx-ΔS188-F196 at a molar ratio of 20:1 (**D**-**F**), and incubated with GFP alone (negative control; **G**-**I**). Note the binding of GFP-pEtx-ΔS188-F196 (arrows in **A** and **C**). Also note that the binding of GFP-pEtx-ΔS188-F196 to the proximal tubules was abolished when co-incubated with an excess of the unlabeled pEtx-ΔS188-F196 (**D**-**F**). No binding to the renal tubules was detected for the GFP alone (**G**-**I**). Nuclei were stained with TO-PRO-3 (blue). Scale bar, 40 μm.

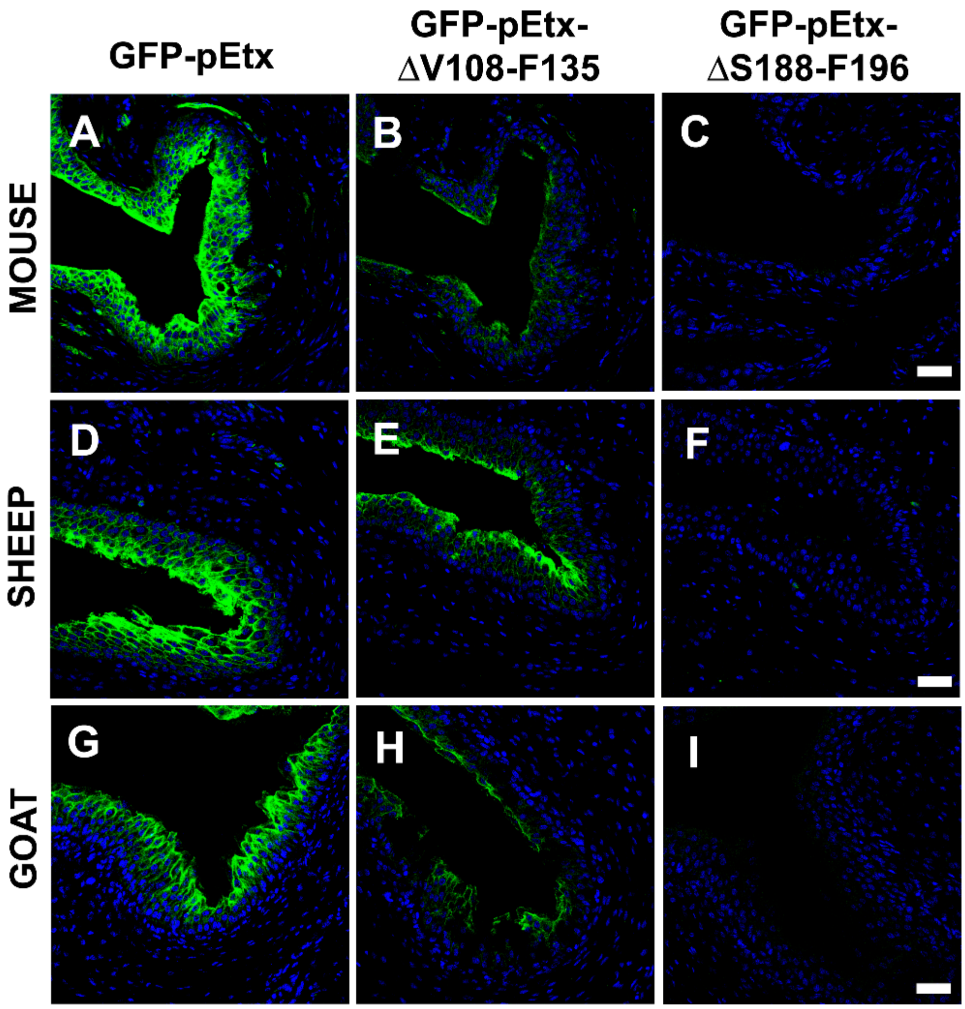

Figure S7. Binding of the GFP-pEtx-ΔV108-F135 and GFP-pEtx-ΔS188-F196 mutants to the urothelium of the bladder. Confocal images of bladder sections from mouse (**A**-**C**), sheep (**D**-**F**), and goat (**G**-**I**) incubated with GFP-pEtx (**A**, **D** and **G**), GFP-pEtx-ΔV108-F135 (**B**, **E** and **H**) or GFP-pEtx-ΔS188-F196 (**C**, **F** and **I**). Note the binding of GFP-pEtx to the urothelium in all the species tested (in green, **A**, **D** and **G**) and the decrease in the binding of GFP-pEtx-ΔV108-F135 to the urothelium (in green, **B**, **E** and **H**). GFP-pEtx-ΔS188-F196 did not bind to the urothelium in any of the species tested (**C**, **F** and **I**). Nuclei were stained with TO-PRO-3 (blue). Scale bar, 40 µm.
